# Inflammation‐Linked Microbiome Alterations Associated with Cognitive Impairment in Older Adults: Insights from the MiaGB Consortium

**DOI:** 10.1002/alz70856_104153

**Published:** 2025-12-25

**Authors:** Sidharth P Mishra, Shalini Jain, Dhananjay Yadav, Lauren Buddendorff, Julia P Hoover, Rohit Shukla, Vivek Kumar, Peter Holland, Michal M Masternak, Corrie Labyak, Cynthia Williams, Adam Golden, Marc Agronin, MiaGB consortium, Hariom Yadav

**Affiliations:** ^1^ Department of Neurosurgery and Brain Repair, Morsani College of Medicine, University of South Florida, Tampa, FL, USA; ^2^ Center for Microbiome Research, Microbiomes Institute, University of South Florida, Tampa, FL, USA; ^3^ FAU Schmidt College of Medicine, Boca Raton, FL, USA; ^4^ University of Central Florida, Orlando, FL, USA; ^5^ Department of Nutrition and Dietetics, Jacksonville, FL, USA; ^6^ University of Central Florida, ORLANDO, FL, USA; ^7^ Miami Jewish Health, Miami, FL, USA; ^8^ University of South Florida, Tampa, FL, USA

## Abstract

**Background:**

The increasing prevalence of cognitive decline and dementia poses a significant public health challenge for older adults, and effective preventive and therapeutic strategies remain elusive. This is largely due to an incomplete understanding of the precise etiology and contributing factors underlying these conditions. Increased systemic inflammation is suspected to elevate the risk of dementia and cognitive decline, yet the causes of chronic inflammation remain poorly understood. Emerging evidence suggests that gut microbiome abnormalities are linked to increased inflammation and a higher risk of dementia. However, it remains unclear whether the rate of cognitive impairment differs with higher systemic inflammation and whether unique microbiome signatures are associated with inflamed cognitive decline and dementia.

**Method:**

Using 165 samples from the Microbiome in Aging Gut and Brain (MiaGB) consortium cohort, systemic inflammatory marker interleukin‐6 (IL‐6) was measured in human plasma via ELISA. Cognitive function was assessed using the Montreal Cognitive Assessment (MoCA) questionnaire, and fecal microbiomes were analyzed through shotgun metagenomic sequencing. Subjects were grouped based on IL‐6 levels (high and low) and cognitive status (normal cognition and cognitive impairment), and their corresponding microbiome signatures were analyzed.

**Result:**

Interestingly, individuals with high IL‐6 levels (IL‐6^High^) exhibited over twice the prevalence of mild cognitive impairment (MCI) compared to those with low IL‐6 levels (IL‐6^Low^) (*n* = 41 IL‐6^High^ vs. 18 IL‐6^Low^). Older adults with low IL‐6 and MCI displayed higher abundances of *Bacteroides*, *Prevotella*, *Alistipes*, *Fusicatenibacter*, and *Parabacteroides*, but lower levels of *Lachnospira*, *Akkermansia*, and *Subdoligranulum* compared to sex‐ and age‐matched cognitively healthy controls with low IL‐6. Conversely, those with high IL‐6 and MCI exhibited higher abundances of *Blautia*, *Prevotella*, and *Fusicatenibacter* and lower abundances of *Lachnospira*, *Akkermansia*, and *Subdoligranulum* compared to IL‐6^High^ controls with normal cognition.

**Conclusion:**

These findings reveal that butyrate‐producing genera such as *Lachnospira*, *Akkermansia*, and *Subdoligranulum* are significantly reduced, while potentially pathogenic *Fusicatenibacter* and commensal *Prevotella* are elevated in individuals with MCI and high IL‐6 levels. These distinct microbial profiles may serve as biomarkers for the early detection of cognitive decline in older adults, highlighting potential targets for therapeutic strategies to preserve brain health during aging.

